# Gender Differences in Antitussive Prescriptions for Chronic Cough in Korea

**DOI:** 10.3390/jcm12227010

**Published:** 2023-11-09

**Authors:** Jinkyeong Park, Yoonki Hong, Ji Young Hong

**Affiliations:** 1 Department of Pulmonary, Allergy and Critical Care Medicine, Kyung Hee University Hospital at Gangdong, School of Medicine, Kyung Hee University, Seoul 05278, Republic of Korea; pjk3318@gmail.com; 2 Department of Internal Medicine, Kangwon National University Hospital, School of Medicine, Kangwon National University, Chuncheon 24289, Republic of Korea; h-doctor@hanmail.net; 3 Division of Pulmonary, Allergy and Critical Care Medicine, Department of Internal Medicine, Chuncheon Sacred Heart Hospital, Hallym University Medical Center, Chuncheon 24253, Republic of Korea; 4 Institute of New Frontier Research Team, Hallym University College of Medicine, Chuncheon 24253, Republic of Korea

**Keywords:** chronic cough, treatment, gender difference

## Abstract

**Background**: We investigated the differences in the characteristics and prognoses between the sexes of patients with chronic cough who were prescribed antitussive agents, using a Korean population-based database. **Methods:** Claims data from South Korea’s Health Insurance Review and Assessment (HIRA) service were analyzed. This retrospective observational cohort study considered chronic cough patients aged 18 years and older who were consistently prescribed antitussive agents for more than 2 months between 1 January 2017 and 30 June 2019. **Results:** Among the 207,989 patients treated for chronic cough, the prevalence of unexplained cough was higher in women (men: 6.2% vs. women: 9.7%) and the prevalence of persistent cough was higher in men (men: 16.8% vs. women: 14.3%). The gap in the proportion of COPD, lung cancer, ILD, GERD, and TB between women and men were largest around the age range of 60–70 years. With the exception of those in their 60s and 70s, women were more likely to have chronic cough and persistent cough than men. Women were more likely to discontinue medication after treatment completion than men. Only 53.9% of patients discontinued cough medication for more than 6 months after treatment completion. Within 12 and 18 months, respectively, 8.9% and 11.9% of them revisited the hospital for chronic cough. Via Cox regression analysis, an age in the 60s or 70s and explained cough were independently associated with a higher risk of revisit for treatment. **Conclusions:** Among patients treated for chronic cough, there were distinct differences in cough characteristics and prescription status between men and women. Our data highlight the need for a new personalized treatment approach to chronic cough, taking into account the gender, age, and underlying diseases of patients. Further research is needed to determine whether appropriate underlying disease control and gender-specific treatment are effective for managing chronic cough.

## 1. Introduction

Coughing, a common activity that prompts hospital visits, is a defense mechanism [1]. However, when it persists for more than 2–3 weeks, it becomes concerning for both patients and physicians. Given the subjectivity of symptom intensity and discomfort, it is challenging to objectively describe severity. The prevalence of chronic cough, defined as a cough lasting over 2 months, is 7.9–12% in adults [2,3,4], similar to that of respiratory diseases such as asthma [5] and COPD [6,7]. While not directly fatal, chronic cough can lead to physical complications and a decline in quality of life [8,9].

Nearly half a century ago, Irwin et al. introduced a systematic approach to identifying (and thus treating) patients with chronic cough using an anatomical diagnostic protocol [10]. Simple interventions, such as discontinuing ACE inhibitors, administering acid-suppressive therapy, and utilizing inhaled steroids, were used to treat these patients. However, clinicians observed that this approach might not be effective for some patients or might lead to cough recurrence in patients who initially responded [11]. This phenomenon was termed cough hypersensitivity syndrome by the European Respiratory Society in 2014 [12] and has sparked further research on chronic cough and the search for new drugs [13,14,15].

Kastelik et al. reported that female patients with chronic cough had more heightened cough reflex sensitivity compared to male patients [16]. Chronic cough, particularly unexplained cough, is common in women, but there are some regional disparities [17,18,19,20,21]. The Rotterdam Study, a large prospective population-based cohort study, showed that two thirds of subjects with persistently unexplained chronic cough and refractory chronic cough were women [22]. On the other hand, the LEAD study from Austria found no sex predominance in chronic cough [23]. The identification of patient-specific characteristics is also important in the selection of candidates for new targets, but the epidemiological data on sex differences in chronic cough characteristics and prognosis are scant.

Korea has implemented a universal health coverage system [24]. Easier primary healthcare access and a simple referral system help chronic cough patients to visit specialist clinics in a relatively short time [25]. The cough patient group and clinical roles between primary clinics and specialist clinics may have some overlap in Korea.

This study evaluated whether the status of antitussive prescriptions for chronic cough differed by gender and other underlying diseases, using a Korean national population-based database. It may provide insight into effective treatment strategies for chronic cough in Korea. 

## 2. Materials and Methods

This study was a retrospective observational cohort analysis that utilized claims data from the Health Insurance Review and Assessment (HIRA) service of Korea. The study cohort included patients aged 18 years and older who received continuous prescriptions for antitussive agents over a period of 2 months between 1 January 2017 and 30 June 2019. Patients older than 100 years were excluded. The study followed these patients until 31 December 2019.

### 2.1. Study Design and Subjects

The study population included patients aged 18 years and above who received consistent prescriptions for antitussives such as codeine, dextromethorphan, levodropropizin, theobromine, and amitriptyline (ATC codes N02AJ09, R05DA04, N02AA08, R05FA, R05DA09, R05FA02, R05DA, R05DB27, and R03DA07), as used in Korea. These medications are based on the Korean Cough Guidelines [26]. The term “explained cough” was used for a cough attributed to conditions with clear diagnostic criteria, while “unexplained cough” referred to a cough that lacked a clear diagnostic attribution [27]. The conditions examined included chronic obstructive pulmonary disease (COPD, ICD-10 codes J43 and J44), asthma (ICD-10 codes J45 and J46), bronchiectasis (ICD-10 code J47), interstitial lung disease (ILD, ICD-10 codes J70, J84, and J98), lung cancer (ICD-10 code C34), pulmonary tuberculosis (TB, ICD-10 codes A15 and A16), gastroesophageal reflux disease (GERD, defined by an endoscopy diagnosis and proton pump inhibitor [PPI] usage), and sinusitis (ICD-10 codes J01 and J32). Cases in which angiotensin-converting enzyme (ACE) inhibitor or dipeptidyl peptidase-4 (DPP-4) inhibitor treatment began within 2 weeks of cough treatment were also investigated. Non-erosive reflux disease (NERD) was defined as the use of PPI medication under the K219 diagnosis code without prior confirmation through upper gastrointestinal endoscopy. In Korea, NERD diagnosis codes are often used as nonspecific diagnosis codes in insurance claims for all gastrointestinal conditions, excluding upper airway cough syndrome, which necessitates PPI therapy but lacks endoscopic evidence. To circumvent this issue, we excluded cases that used NERD codes without pursuing an endoscopic diagnosis from the “explained cough” category. A persistent cough was defined as a chronic cough lasting for 1 year despite antitussive treatment. We examined the duration of the discontinuation of cough medication after the end of treatment, with the observed periods ranging from 4 months to 2 years at the specific intervals of 4, 6, 9, 12, 18, and 24 months. The likelihood of patients revisiting the doctor for cough-related issues was investigated relative to the time elapsed since the discontinuation of the cough medication.

### 2.2. Data Source

The National Health Insurance (NHI) system of South Korea is a single-payer, public system that has covered the South Korean population since 1989. The NHI covers 97% of the population, while the Medical Aid program covers the remaining 3%. All claims data submitted by the NHI and the Medical Aid program are reviewed by the HIRA.

### 2.3. Ethics

The research protocol for this study was approved by the Health Insurance Review and Assessment (HIRA) service (M20220714005). The Kyung Hee University Hospital at Gangdong Institutional Review Board waived the requirement for ethics approval (KHNMC 2022-08-029), given that this study used previously collected data. The study was conducted in accordance with the Declaration of Helsinki.

### 2.4. Statistical Analysis

This study involved the procurement of population estimates by age and year from the National Statistical Office. The primary endpoint of interest was the discontinuation rate of cough medication, while the secondary endpoint was the revisit rate within 18 months. To effectively control for different age distributions, we computed the age-adjusted incidence rates of chronic cough for the standard Korean population from 2017 to 2018 using the direct method [28]. Patient characteristics and variables were presented by sex, either as percentages or as medians with interquartile ranges (IQRs). Depending on the characteristics of these groups, we used suitable statistical tests for comparisons, such as the *t*-test or chi-square test. To examine the risk factors associated with revisit for treatment, we used Cox regression, adjusting for potential confounding variables. All statistical analyses were performed using R version 3.4.4 (R Foundation for Statistical Computing, Vienna, Austria), with the significance level set at *p* < 0.05.

## 3. Results

### 3.1. Characteristics of Study Participants 

During the study period, 207,989 patients in Korea were prescribed with antitussive agents, representing 0.50% of the total adult population. Table 1 illustrates the characteristics of the study subjects divided by sex. The group with the highest prevalence of chronic cough comprised individuals in their 70s. The number of patients suffering from chronic cough increased with age until the 70s. Although women had a higher incidence of chronic cough in most age groups, a shift in the sex ratio was observed in individuals in their 60s and 70s. The most common cause was bronchial asthma (74.1%), followed by NERD (73.6%), and sinusitis (51.6%). Men with chronic cough were more likely to have COPD, lung cancer, ILD, TB, and GERD, while NERD was more common in women with chronic cough. Prior use of ACE inhibitors and DPP-4 inhibitors was more common in men.

The prevalence of unexplained cough without an identifiable cause and persistent cough was 8% and 15.5%, respectively. Women had a significantly higher rate of unexplained cough than men (9.7% vs. 6.2%, *p* < 0.001), while men had a higher rate of persistent cough than women (16.8% vs. 14.3%, *p* < 0.001).

### 3.2. Differences in Chronic Cough Characteristics by Sex and Age

**Figure 1:**
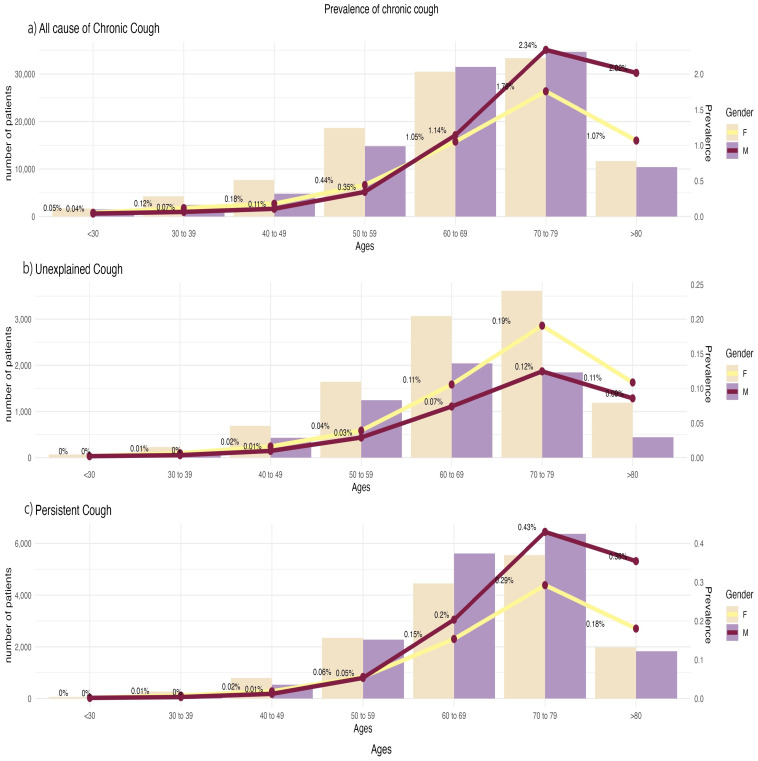
The prevalence of al-cause (**a**), unexplained (**b**), and persistent (**c**) chronic cough, according to age, in Korea.

**Figure 2:**
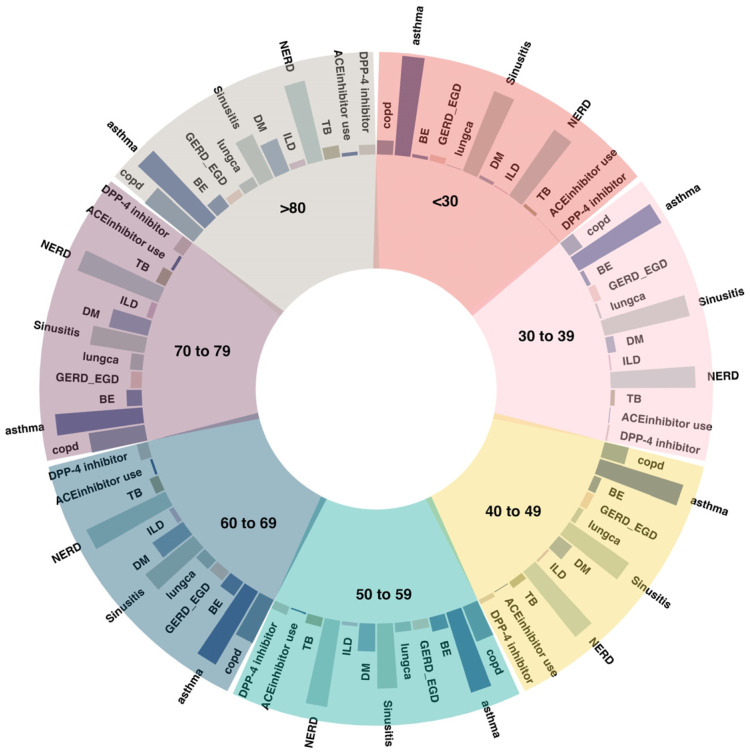
Comorbid conditions associated with chronic cough by age.

The sex differences in chronic cough become more pronounced when the age distributions are considered (Figure 1). Both chronic cough and persistent cough were more common in men in their 60s and 70s, while women were more affected in other age groups (Figure 1). Unexplained cough, however, was more common in women across all age groups. These results can be explained via an examination of the distribution of underlying diseases by age (Figure 2). The rates of asthma and rhinitis were similar across all age groups (>70% and >80%, respectively). Conversely, the incidences of COPD, ILD, and TB increased with age, and the proportions of patients with lung cancer and GERD were highest in people aged 60–70 years. In addition, the discrepancy in the proportion of COPD, lung cancer, ILD, GERD, and TB between women and men broadened around the age of 60–70 years. The reason why chronic cough and persistent cough increased in men in their 60s and 70s may be because the high smoking rate and different lifestyle habits among men have led to an increase in cough-related diseases, such as pulmonary parenchymal disease and GERD.

### 3.3. Treatment Administered to Study Participants

Patients with chronic cough consumed antitussive agents for a median duration of 110.00 days (IQR: 76.0–222.0). Men required medication for a longer period than women (median: 116 days vs. 104 days, *p* < 0.001). In instances of persistent cough, the proportion of unexplained cough was also higher in women (10.5%) than in men (5.7%, *p* < 0.001). Amitriptyline and theobromine formulations were frequently employed to manage coughing. Although no significant differences were observed in the use of codeine, dextromethorphan, and inhaled corticosteroids (ICSs) between men and women, amitriptyline, antihistamines, leukotriene receptor antagonists (LTRAs), proton pump inhibitors (PPIs), and theobromine were more commonly used by men (Table 2).

### 3.4. Long-Term Outcomes of Chronic Cough

Table 3 depicts the drug discontinuation rates of patients who required antitussive treatment for chronic cough in Korea. Among these patients, 62.3% had not taken antitussives for more than 4 months, 53.9% for more than 6 months, and 36.9% for more than 1 year. Women were more likely to discontinue antitussive agents than men across various drug withdrawal periods. Men with explained cough were significantly less likely to cease medication than those with unexplained cough. Conversely, no difference was observed between explained and unexplained cough in women with a duration of drug discontinuation of less than 12 months. Women who discontinued antitussive agents for more than 12 months exhibited a higher proportion of unexplained cough than explained cough, similar to men.

**Figure 3:**
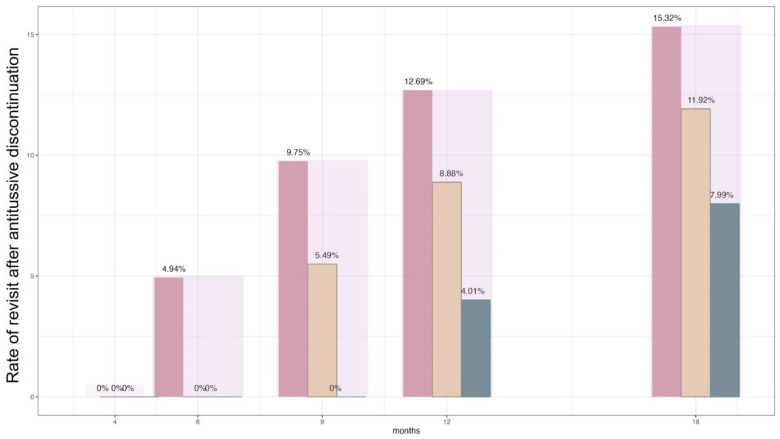
Rate of revisit after treatment completion for chronic cough. The diagram illustrates the proportion of patients who returned for a follow-up visit over time, grouped according to their periods of antitussive discontinuation. The pink bar represents patients who discontinued medication for at least 4 months, the mustard-colored bar corresponds to those with a discontinuation period of at least 6 months, and the green bar signifies patients who enjoyed a discontinuation period of 9 months or longer.

Figure 3 presents the rate of revisit for cough in patients who discontinued antitussive agents for a certain period post treatment. Of the patients who discontinued antitussive agents for over 6 months, 5.49%, 8.88%, and 11.92% revisited the hospital for chronic cough at 9 months, 12 months, and 18 months, respectively. Intriguingly, as the remission duration extended, the rate of patient revisit tended to decrease. At the 12-month follow-up after treatment completion, approximately 12.69% of patients who discontinued medication for 4 months revisited the hospital, while the rate decreased to 8.88% and 4.01% for those who sustained 6-month and 9-month discontinuation periods, respectively. 

Table 4 shows that the Cox regression analysis adjusting for a multivariable model revealed a significantly increased risk of revisit in patients who discontinued medication for over 6 months in their 60s (HR, 2.2; 95% CI, 1.85–2.60, *p* < 0.001) and 70s (HR, 2.2; 95% CI, 1.86–2.61, *p* < 0.001) compared to those in their 30s. Also, the hazard ratio for patients with unexplained cough is 0.7 (95% CI 0.65–0.75) compared to those with explained cough. The longer the duration of antitussive medication, the higher the rate of revisit after the end of treatment. Similar results were observed in the patients with different discontinuation periods.

## 4. Discussion

This study examined the prevalence, characteristics, and prescription status of chronic cough patients with antitussive agents using a national population-based database. Our findings confirm that chronic cough, requiring treatment for over 2 months, affects 0.5% of Korean adults, with the incidence increasing with age. In addition, we found significant disparities in prevalence across sex and age groups. These observations highlight the importance of considering such demographic differences in the diagnosis and management of chronic cough. Furthermore, these insights provide valuable contributions to the collective effort to enhance understanding and develop more effective treatments for chronic cough.

There are notable disparities among studies regarding the epidemiology of chronic cough, mainly due to the varying definitions of the condition [4]. Importantly, the reported prevalence of chronic cough exhibits significant geographical variation. Specifically, the prevalence in Asia is typically below 5%, considerably lower than that reported in Europe, America, and Australia, which ranges between 10% and 20% [29]. The relatively lower prevalence in our study can be largely attributed to the unique criteria we employed, focusing on patients who required medication treatment for more than two consecutive months. This approach contrasts with those in previous studies that have typically relied on patient interviews based on recollections. The prevalence may have been somewhat underestimated, as the study cohort likely had a coughing period prior to the initiation of dosing. Moreover, many previous studies have focused on patients who frequently visit cough clinics, potentially introducing a high degree of selection bias associated with cough-related cases. These considerations should be taken into account when comparing the prevalence rates across different studies.

The conditions causing cough identified in our study align with those reported in previous studies [30,31]. Bronchial asthma and NERD were the prevalent causes of chronic cough in our study. Interestingly, chronic cough often cooccurs with conditions such as COPD, lung cancer, TB, and GERD in men across all age groups. Consistent with earlier studies, our data demonstrated a strong correlation between NERD and chronic cough in women of all ages [32]. Men had higher rates of explained cough and overall chronic cough, specifically in their 60s and 70s, while women had higher rates of unexplained cough across all ages. These findings suggest a significant role of smoking, along with inherent biological sex differences, in chronic cough [16,33]. There are more male than female smokers in Korea, resulting in a higher incidence of respiratory-related illnesses—closely linked with smoking—among individuals in their 60s and 70s. Reinforcing previous research [34,35,36], our study suggests that persistent cough often originates from explained cough. In the work by Koskela et al., almost half the individuals with chronic cough still suffered from the condition 5 years after their initial diagnosis, linking this persistent cough to underlying conditions such as chronic rhinitis, esophageal reflux disease, and bronchial hyperresponsiveness [34]. These findings underscore the complexity of chronic cough, advocating for an individualized, comprehensive approach to its diagnosis and management.

This study unveiled fascinating insights regarding the efficacy of antitussive treatment in patients suffering from chronic cough. Remarkably, approximately half of the patients were unable to maintain drug discontinuation within 6 months of treatment completion, indicating a significant low discontinuation rate. Also, a sizable number of patients revisited the doctor for cough-related issues following several months of discontinuation. These results are consistent with previous studies suggesting the need for a novel antitussive agent for patients with chronic refractory cough [14,37]. 

One of the most notable findings concerned the gender disparities observed in chronic cough outcomes. Women demonstrated higher discontinuation rates after treatment completion, potentially due to the higher incidence of unexplained cough in women than in men. While the discontinuation rates after treatment completion were higher in unexplained cough than in explained cough in men, the discontinuation rates in women were similar for both categories. Whether these results stem from superior drug treatment efficacy in controlling symptoms in women compared to men, or suggest inadequate medication adherence, remains unclear. However, given the previous studies showing that patients with unexplained chronic cough or refractory chronic cough are extremely difficult to control and are less responsive to several medications [38,39], it is possible that women or men with unexplained cough were quicker to give up the ineffective treatment despite cough persistence, leading to a false higher remission rate. These findings underscore the need for a multifaceted personalized approach that considers the clinical heterogeneity of the chronic cough population.

Chronic cough management includes pharmacological and non-pharmacological therapy. The effectiveness of pharmacological therapies is limited in refractory cough and unexplained cough [40,41]. Non-pharmacological therapy is effective for chronic cough with fewer side effects, whether alone or combined with pharmacological therapies, but there is a paucity of clinical data [2,42]. Interestingly, several studies have reported that oscillatory positive expiratory pressure therapy in patients with COPD with cough improved their cough-related quality of life and reduced the frequency of coughs [43,44].

Further research is warranted to accurately identify the appropriate groups that will benefit from conventional antitussives and to evaluate the efficacy of novel treatment strategies using pharmacological and non-pharmacological approaches for each specific patient group. 

This study had several limitations. First, our data were from nationwide insurance claims from a single country. While the general treatment approach might be comparable across nations, variations in medication use, insurance coverage, and healthcare utilization patterns are present. Therefore, the corroboration of these findings with data from other countries could offer more generalizable conclusions. Second, our definition of chronic cough was strictly based on the duration of medication use as recorded in the insurance claims data. This methodology may have imposed stringent criteria for selecting patients with chronic cough, as periods without medication use were not included in the definition. Third, insurance claims data inherently lack information about lifestyle or quality of life. Although smoking is a significant cause of cough, we were unable to identify or adjust for its effect. We only identified groups with a high prevalence of conditions known to be caused by or closely related to smoking. Future studies should explore this issue using more detailed data. Also, it is unknown whether the medication discontinuation was achieved via the effect of antitussives or through therapy specific to the etiologies in explained cough. Despite these limitations, our results provide valuable insights into the epidemiology, characteristics, and treatment outcomes of chronic cough in men and women. Our findings can be used to inform clinical practice and guide the development of more effective and targeted interventions for chronic cough. Further research is needed to determine whether appropriate underlying disease control and gender-specific treatments are effective for managing chronic cough.

## 5. Conclusions

This study offers invaluable insights into the prescription status of antitussive drugs for chronic cough patients within the Korean population. Importantly, we identified significant gender disparities in the underlying causes and rate of revisit. Our findings can inform clinical practice and guide the creation of targeted interventions for chronic cough, emphasizing the need for a new, personalized approach that takes into account sex differences and individual patient traits.

## Figures and Tables

**Table 1:** Baseline characteristics of study subjects.

	Total	Men	Women	*p*-Value
	207,989	100,155	107,834	
Age (median [IQR])	69.0 [60.0–76.0]	69.0 [61.0–76.0]	68.0 [58.0–76.0]	<0.001
Ages (%)				<0.001
<30	3215 (1.5)	1508 (1.5)	1707 (1.6)	
31 to 40	6716 (3.2)	2457 (2.5)	4259 (3.9)	
41 to 50	12,507 (6.0)	4800 (4.8)	7707 (7.1)	
51 to 60	33,459 (16.1)	14,812 (14.8)	18,647 (17.3)	
61 to 70	61,984 (29.8)	31,498 (31.4)	30,486 (28.3)	
71 to 80	68,011 (32.7)	34,668 (34.6)	33,343 (30.9)	
>80	22,097 (10.6)	10,412 (10.4)	11,685 (10.8)	
COPD (%)	84,652 (40.7)	55,654 (55.6)	28,998 (26.9)	<0.001
Asthma (%)	154,160 (74.1)	74,069 (74.0)	80,091 (74.3)	0.099
Bronchiectasis (%)	25,790 (12.4)	12,503 (12.5)	13,287 (12.3)	0.266
EGD-confirmed reflux (%)	17,973 (8.6)	9743 (9.7)	8230 (7.6)	<0.001
Lung cancer (%)	19,122 (9.2)	13,521 (13.5)	5601 (5.2)	<0.001
Sinusitis (%)	107,379 (51.6)	49,489 (49.4)	57,890 (53.7)	<0.001
Diabetes mellitus (%)	59,707 (28.7)	31,407 (31.4)	28,300 (26.2)	<0.001
Interstitial lung disease (%)	8880 (4.3)	5369 (5.4)	3511 (3.3)	<0.001
NERD (%)	153,138 (73.6)	70,992 (70.9)	82,146 (76.2)	<0.001
Pulmonary tuberculosis (%)	17,306 (8.3)	10,769 (10.8)	6537 (6.1)	<0.001
ACE inhibitor use (%)	4283 (2.1)	2557 (2.6)	1726 (1.6)	<0.001
DPP-4 inhibitor use (%)	17,793 (8.6)	9485 (9.5)	8308 (7.7)	<0.001
Bronchoscopy (%)	97 (0.0)	65 (0.1)	32 (0.0)	<0.001
pH monitoring (%)	65 (0.0)	29 (0.0)	36 (0.0)	0.655
Unexplained cough	16,731 (8.0)	6223 (6.2)	10,508 (9.7)	<0.001
Persistent cough	32,302 (15.5)	16,842 (16.8)	15,460 (14.3)	<0.001
Cough duration (median [IQR])	110.0 [76.00–222.0]	116.0 [78.0–244.0]	104.0 [74.0–203.0]	<0.001

ACE = angiotensin-converting enzyme, COPD = chronic obstructive pulmonary disease, DPP-4 = dipeptidyl peptidase-4, EGD = esophagogastroduodenoscopy, NERD = non-erosive reflux disease.

**Table 2:** Medication prescriptions according to gender.

	Overall	Men	Women	*p*-Value
Codeine	48,436 (63.0)	23,317 (62.9)	25,119 (63.1)	0.668
Dextromethorphan	1427 (61.3)	582 (60.6)	845 (61.8)	0.585
Theobromine	58,582 (67.3)	29,107 (67.9)	29,475 (66.8)	<0.001
First-generation histamine	62,607 (31.8)	30,759 (32.9)	31,848 (30.8)	<0.001
Second-generation histamine	67,887 (37.0)	32,794 (37.7)	35,093 (36.4)	<0.001
Inhaled corticosteroid	52,718 (53.1)	25,589 (53.0)	27,129 (53.2)	0.464
Leukotriene antagonist	54,191 (52.8)	25,043 (53.5)	29,148 (52.1)	<0.001
Proton pump inhibitor	72,882 (41.6)	35,188 (42.5)	37,694 (40.7)	<0.001
Amitriptyline	60,384 (77.1)	26,242 (78.3)	34,142 (76.2)	<0.001

**Table 3:** The drug discontinuation rate in chronic cough patients treated with antitussive agents.

	Total	Men		*p*-Value ^∫^	Women		*p*-Value ^¶^	*p*-Value ^∫^
		Explained Cough	Unexplained Cough		Explained Cough	Unexplained Cough		
No. of patients	207,989	93,932	6223		97,326	10,508		
For 4 months	129,519 (62.3)	56,938 (60.6)	3990 (64.1)	<0.001	61,939 (63.6)	6652 (63.3)	<0.001	0.502
For 6 months	112,166 (53.9)	49,251 (52.4)	3497 (56.2)	<0.001	53,558 (55.0)	5860 (55.8)	<0.001	0.152
For 9 months	93,238 (44.8)	40,800 (43.4)	2995 (48.1)	<0.001	44,530 (45.8)	4913 (46.8)	<0.001	0.052
For 12 months	76,667 (36.9)	33,307 (35.5)	2525 (40.6)	<0.001	36,684 (37.7)	4151 (39.5)	<0.001	<0.001
For 18 months	41,209 (19.8)	17,760 (18.9)	1433 (23.0)	<0.001	19,626 (20.2)	2390 (22.7)	<0.001	<0.001
For 24 months	16,043 (7.7)	6735 (7.2)	586 (9.4)	<0.001	7789 (8.0)	933 (8.9)	<0.001	0.002

Number (percentage). ^∫^ *p* value between explained cough and unexplained cough. ^¶^ *p* value between men and women.

**Table 4:** Cox regression analysis of the rate of revisit for antitussive treatment for chronic cough.

	Remission for 4 Months			Remission for 6 Months			Remission for 9 Months		
Variables	Hazard Ratio	95% CI	*p* Value	Hazard Ratio	95% CI	*p* Value	Hazard Ratio	95% CI	*p* Value
Age									
<30	1			1			1		
31–40	1.2883	1.0912–1.5210	0.0028	1.3396	1.0997–1.6319	0.0037	1.2413	0.9588–1.607	0.1008
41–50	1.7244	1.4809–2.0079	<0.001	1.6864	1.4056–2.0234	<0.001	1.5415	1.2156–1.955	<0.001
51–60	1.9351	1.6744–2.2364	<0.001	1.8815	1.5827–2.2366	<0.001	1.7124	1.3683–2.143	<0.001
61–70	2.2003	1.9077–2.5376	<0.001	2.1926	1.8494–2.5996	<0.001	1.9917	1.5975–2.483	<0.001
71–80	2.2521	1.9530–2.5971	<0.001	2.2025	1.8579–2.6110	<0.001	2.041	1.6381–2.545	<0.001
>80	1.8808	1.6225–2.1803	<0.001	1.8197	1.5248–2.1716	<0.001	1.6064	1.2759–2.023	<0.001
Gender									
Men	1			1			1		
Women	1.0312	1.0026–1.0605	0.032	1.0361	1.0012–1.0721	0.0423	1.0387	0.9912–1.088	0.1117
Unexplained cough	0.7347	0.6942–0.7776	<0.001	0.6976	0.6503–0.7484	<0.001	0.6547	0.5937–0.722	<0.001
Anittussive duration	1.0008	1.0006–1.0009	<0.001	1.0005	1.0003–1.0006	<0.001	1.0004	1.0002–1.001	<0.001

## Data Availability

We cannot share the administrative data of the Korean government.
